# Academy of Medicine Notes

**Published:** 1893-11

**Authors:** 


### Academy of Medicine Notes.

Dr. H. U. Williams has resigned as curator of the museum, and Dr. F. S. Metcalfe has been appointed to fill the vacancy.

---

The Section on Medicine will meet November 14th. The subject to be discussed will be Stomach Affections and their Treatment.

---

The next stated meeting of the Academy will be held Tuesday, December 5, 1893, under the auspices of the Section on Surgery.

THE Section on Anatomy, Physiology, and Pathology did not meet on October 17th, but will meet on Tuesday, October 31, 1893. Papers will be read by Drs. Bennett and Benedict.

---

THE council has recommended to invite the medical students to the meetings of the Academy, subject to the approval of the Academy. The matter will be presented at the December meeting.

---

THE Section on Surgery will hold its next meeting November 7th. The following programme has been prepared for the evening: The Surgery of the Thorax, Dr. John Parmenter; Thoracic Diseases of Children, Dr. Irving M. Snow; Thoracic Diseases in the Adult, Dr. DeLancy Rochester.

